# Prevalence of smear positive pulmonary tuberculosis among prisoners in North Gondar Zone Prison, northwest Ethiopia

**DOI:** 10.1186/1471-2334-12-352

**Published:** 2012-12-15

**Authors:** Beyene Moges, Bemnet Amare, Fanaye Asfaw, Wogahta Tesfaye, Moges Tiruneh, Yeshambel Belyhun, Andargachew Mulu, Afework Kassu

**Affiliations:** 1 Department of Immunology and Molecular Biology, School of Biomedical and Laboratory Sciences, College of Medicine and Health Sciences, University of Gondar, P.O.BOX 196, Gondar, Ethiopia; 2 Department of Medical Biochemistry, College of Medicine and Health Sciences, University of Gondar, Gondar, Ethiopia; 3 Department of Microbiology, School of Biomedical and Laboratory Sciences, College of Medicine and Health Sciences, University of Gondar, Gondar, Ethiopia; 4 Department of Pathology, College of Medicine and Health Sciences, University of Gondar, Gondar, Ethiopia; 5 Institute of Virology, Medical Faculty, University of Leipzig, Leipzig, Germany

**Keywords:** Prison, Tuberculosis, TB/HIV co-infection, Ethiopia

## Abstract

**Background:**

People concentrated in congregated systems, such as prisons, are important but often neglected reservoirs for TB transmission, and threaten those in the outside community. Therefore, this study was conducted to determine the prevalence of tuberculosis in a prison system of North Gondar Zone.

**Methods:**

An active case-finding survey in North Gondar Prison was carried out from March to May 2011. All prison inmates who had history of cough for at least a week were included in the study. Three morning sputum samples were collected from suspected inmates and examined through fluorescence microscopy. Fine needle aspiration cytology was done for those having significant lymphadenopathy. Pre and post HIV test counseling was provided after written consent. Binary logistic and multivariable analysis was performed using SPSS version 16.

**Results:**

A total of 250 prisoners were included in the survey. Among these, 26 (10.4%) prisoners were found to have TB giving a point prevalence of 1482.3 per 100,000 populations of smear positive TB among the TB suspects. All the inmates who participated in the study volunteered for HIV testing and a total of 19(7.6%) inmates were found to be reactive for the HIV antibody test amongst of which 9(47.4%) had TB co-infection. The prevalence of HIV infection in the TB infected inmates was found to be 34.6% (9/26). From the 26 TB cases identified 12 (46.2%) were having under nutrition (BMI < 18.5kg/m^2^).

**Conclusions:**

There is high prevalence of TB in North Gondar Prison with possible active transmission of TB within the prison. There was a high prevalence of HIV among the TB suspects. Strong cooperation between prison authorities and the national tuberculosis control programmes is urgently required to develop locally appropriate interventions to reduce transmission. The determinants for poor nutrition in the prison need also further investigation.

## Background

The conditions in developing countries make tuberculosis (TB) a major problem in prisons. TB is endemic in the general population, and detainees often come from underprivileged communities with higher risk for TB. Prison cells are often poorly ventilated and house dozens of detainees who, in many prisons, mix all day long with detainees from other cells in enclosed spaces [1]. On top of this, the prisons’ health system is inadequate in almost all developing countries and, particularly in Sub-Saharan Africa (SSA). To make matters worse, prison populations have high HIV (human immune deficiency virus) sero-prevalences [1].

Despite the fact that the global focus on TB control is on early diagnosis and treatment of people in high TB and TB/HIV-endemic countries, people in prisons are often neglected reservoirs for TB transmission threatening those in the outside community [2].

Most TB surveys in prisons showed high TB prevalence rates ranging from 156.2/100,000 to 6500/100,000 [2-6]. As expected, there was a very high rate of HIV co-infection in many of the studies, ranging from 26% in Tanzania to 73% in Malawi [7,8]. Generally, the prevalence of TB in SSA prisons is estimated to be 6–30 times higher than that in the general population [9]. The TB prevalence rate in Zambian prisons in one study was about 10 times that of the outside population [10].

The point prevalence of pulmonary TB in three major prison settings of Eastern Ethiopia was 1913 per 100,000, about seven times higher than that of the general population [11]. As there are no studies conducted in the prisons of the Northwestern part of Ethiopia, this study was planned to investigate the prevalence of TB among prison inmates and the risk factors associated with increased transmission in this area.

## Methods

### Study design, area and period

A cross sectional study was conducted in North Gondar Zone Prison between March and May 2011 to determine the prevalence of TB. The prison is located in the historic city of Gondar. It accommodates thousands of inmates every year and is one of the major prisons in North Gondar Zone. It had 1754 inmates during this survey. The prison has a clinic for the prisoners and the prison staff. It is meant for the diagnosis and treatment of infectious and non infectious diseases. It also provides service for the diagnosis and treatment of TB and HIV.

### Specimen collection and processing

A structured and pretested questionnaire was used to collect socio-demographic and relevant clinical data of the study subjects by physicians.

#### Sputum microscopy

Three morning sputum samples were collected from patients having cough of at least 1 week duration. Sputum-smear microscopy using a light emitting diode (LED) fluorescence microscopy was done at Gondar University Hospital Laboratory on the day of collection following the manufacturer’s procedures (PARTEC GmbH). Two slides were stained for every sample and the slides were read by two experienced microbiologists separately. Whenever there were discordant results, a third expert microbiologist read the slides and the report of the third reader was taken as final. Smear positivity was defined as the presence of at least one positive smear result using the LED microscopy; this was used to put them on treatment.

#### Fine needle aspiration cytology

All the TB suspects were screened for the presence of lymphadenopathy both subjectively and objectively by physicians. Fine needle aspiration cytology (FNAC) was performed by a pathologist for patients having significant lymphadenopathy (lymph nodes greater than 1x1cm in size). The slides were stained with Wright stain and TB was diagnosed by the presence of epitheloid granuloma and necrosis. The results were read by an experienced pathologist at the University of Gondar, Department of Pathology.

#### Blood collection, serum separation and HIV serology

To determine the HIV serostatus of the TB suspected inmates, pre-test counseling was provided to the volunteer prisoners by a trained physician. Whole blood was collected from prison inmates. Serum was separated by centrifugation within 2h of collection and kept at -20°C until used. The presence of HIV antibodies was determined by an enzyme-linked immunosorbent assay (ELISA) following the manufacturer’s instruction (Vironostica HIV Uni-Form II plus O, Organon Teknika, Boxtel, the Netherlands). After testing, the prisoners were provided with post testing counseling by the counselor. To assure confidentiality of test results, only code numbers were used to identify serum of prisoners.

#### Nutritional assessment

Body weight was determined to the nearest 0.1 kg on an electronic digital scale and height was measured to the nearest 0.1 cm. Body mass index (BMI), defined as the weight in kilogram of the individual divided by the square of the height in meter, was used to determine the nutritional status of the patients into severe malnutrition (BMI < 15.9 kg/m^2^), moderate malnutrition (BMI = 16–16.9 kg/m^2^), mild malnutrition (BMI = 17–18.4 kg/m^2^) and normal (BMI = 18.5-25 kg/m^2^) as recommended by WHO [12].

### Statistical analysis

The collected data was computerized using excel program, cleaned and exported to be analyzed by SPSS version 16 (SPSS, Inc., Chicago, IL, USA). Means and standard deviations were calculated for continuous variables using this software while crude and adjusted Odds ratios (OR) were calculated to check statistical association between the dependent and independent variables using the binary logistic regression and multivariable logistic regression models. All variables of the study were initially tested for association with smear positivity by using the binary logistic regression model. Those which showed statistical association with sputum positivity by the logistic regression model were put in the multivariable analysis model to check if the association existed after controlling against all the rest of the variables.

### Ethical issue

The study was conducted after ethical approval was obtained from Institutional Review Board of the University of Gondar and after informed consent was obtained from study subjects. Positive patients for TB and/or HIV were treated by the prison staff following the nation’s standard clinical management protocols.

## Results

There were a total of 250 prison inmates with cough of more than one week duration and all were included in the survey. Table 1 shows distribution of TB positivity among different sociodemograhic characteristics of the prison inmates. A total of 26 (10.4%) inmates were found to have TB during the survey using LED microscopy. Three of them also had lymph node TB. The point prevalence of TB in the prison was then calculated to be 1482.3 per 100,000 populations among the TB suspects. The mean number of prisoners per cell was 333 (±126.5, 5–500). In line with this, it was found out that almost all (96%) of the TB cases shared a single cell with more than 200 other inmates. On the other hand the average duration of stay in the prison was 3 years with majority (65.4%) of the TB positive inmates left to stay for more than a year. Staying for 2–6 months in the prison was found to be associated with TB positivity (Crude OR = 0.362, 95%CI = 0.145-0.907). However, this association with TB positivity was not observed when length of stay in the prison was adjusted against other socio-demographic characteristics (OR = 0.651, 95%CI = 0.405-1.047). The age and educational status of the inmates and number of prisoners per cell were not found to be statistically associated with TB positivity (Table 1).

**Table 1:** TB positivity as detected by LED microscopy among different socio-demographic factors of prison inmates in North Gondar Zone Prison, using logistic and multivariable logistic regression analysis, N = 250

**Variables**	**No. tested (%)**	**No. positive (%)**	**No. negative (%)**	**Crude OR**	**95%CI**	**Adjusted OR**	**95%CI**
**Age (in years)**						0.792	0.537-1.170
0-20	31(12.4)	2(6.5)	29(93.5)	0.906	0.076-10.788		
21-30	108(43.2)	15(13.9)	93(86.1)	0.388	0.48-3.141		
31-40	51(20.4)	6(11.8)	45(88.2)	0.469	0.52-4.199		
41-50	31(12.4)	2(6.5)	29(93.5)	0.906	0.076-10.788		
>50	29(11.6)	1(3.4)	28(96.6)	Ref			
**Educational status**						1.247	0.643-2.417
Uneducated	132(52.8)	15(11.4)	117(88.6)	-	-		
primary education	94(37.6)	7(7.4)	87(92.6)	-	-		
secondary education	22(8.8)	4(18.2)	18(81.8)	-	-		
Tertiary education	2(0.8)	0(0)	2(100)				
**Length of stay in the prisons (in months)**						0.651	0.405-1.047
<2	18(7.2)	1(5.6)	17(94.4)	1.382	0.166-11.483		
2-6	60(24)	11(18.3)	49(81.7)	0.362	0.145-0.907		
6-12	39(15.6)	4(10.3)	35(89.7)	0.711	0.210-2.407		
>12	133(53.2)	10(7.5)	123(92.5)	Ref			
**Number of prisoners per cell**						1.249	0.802-1.946
1-100	24(9.6)	0(0)	24(100)	2.454	-		
101-200	6(2.4)	1(16.7)	6(83.3)	0759	0.082-7.072		
201-300	61(24.4)	8(13.1)	53(86.9)	1.006	0.385-2.628		
301-400	68(27.2)	5(7.4)	63(92.6)	1.914	0.641-5.718		
401-500	91(36.4)	12(13.2)	79(86.8)	Ref			

*TB* = tuberculosis; *OR* = odds ratio; *CI* = confidence interval; *LED* = light emitting diode; Ref = reference variable.

Table 2 illustrates the distribution of TB against duration of cough, timing of cough occurrence, history of previous treatment for TB, nutritional status and HIV sero-status among the prison inmates. Majority (84.6%) of the inmates with TB reported a cough of more than 2 months duration with twenty (76.9%) of them developed the cough after imprisonment. Five (19.2%) of the TB cases had history of previous treatment for TB. The prevalence of history of previous treatment among those who were not found to have smear positive pulmonary TB was 22/224(9.8%). The mean (±SD, range) BMI in kg/m^2^ of the prisoners with TB was 19.4 (±2.5, 15.6-24.8). From the 26 TB cases identified 12 (46.2%) were having under nutrition (BMI < 18.5kg/m^2^).

**Table 2:** TB positivity as detected by LED microscopy among different clinical factors of prison inmates in North Gondar Zone Prison, using logistic and multivariable regression analysis, N = 250

**Variables**	**No. tested (%)**	**No. positive (%)**	**No. negative (%)**	**Crude OR**	**95%CI**	**Adjusted OR**	**95%CI**
**Duration of Cough**						1.471	0.946-2.286
1 week	17(6.8)	1(5.9)	16(94.1)	0.444	0.283-17.749		
2 weeks	14(5.6)	0(0)	14(100)	0.999	-		
3 weeks	4(1.6)	0(0)	4(100)	0.999	-		
4 weeks	19(7.6)	2(10.5)	17(89.5)	0.823	0.258-5.509		
8 weeks	17(6.8)	1(5.9)	16(94.1)	0.444	0.283-17.749		
>8 weeks	179(71.6)	22(12.3)	157(87.7)				
**Time of occurrence of the cough**						1.332	0.416-4.262
Before imprisonment	51(20.4)	6(23.1)	45(20.1)	0.838	0.318-2.209		
After imprisonment	199(79.6)	20(76.9)	179(79.9)				
**Nutritional status**						0.383	0.154-0.954
Under nutrition (BMI < 18.5kg/m^2^)	69(27.6)	12(17.4)	57(82.6)	0.398	0.174-0.911		
Normal nutrition (BMI > 18.5kg/m^2^)	181(72.4)	14(7.7)	167(92.3)				
**History of previous treatment**						0.469	0.128-1.726
Yes	27(10.8)	5(18.5)	22(81.5)	0.457	0.157-1.334		
No	223(89.2)	21(9.4)	202(91.6)				
**HIV status of the inmates**						0.082	0.026-0.258
Positive	19(7.6)	9(47.4)	10(52.6)	0.088	0.032-0.247		
Negative	231(92.4)	17(7.4)	214(92.6)				

*BMI* = body mass index; *TB* = tuberculosis; *OR* = odds ratio; *CI* = confidence interval; *LED* = light emitting diode.

All the inmates who participated in the study volunteered for HIV testing and a total of 19 (7.6%) were found to be reactive for the HIV antibody test amongst of which 9 (47.4%) had TB co-infection. The prevalence of HIV infection in the TB infected inmates was calculated to be 34.6%. HIV positivity and under nutrition were significantly associated with TB positivity (Crude OR = 0.398, 95%CI = 0.174-0.911 and OR = 0.088, 95%CI = 0.032-0.247). The association of under nutrition and HIV sero-reactivity with TB positivity was also observed when adjusted OR was calculated using multivariable logistic regression model (OR = 0.383, 95%CI = 0.154-0.954 and OR = 0.082, 95%CI = 0.026-0.258, respectively). Duration of cough, timing of cough occurrence and history of previous treatment for TB were not found to be associated with TB positivity (Table 2).

## Discussion

Prevalence rates of TB in prisons usually exceed the rates in the general population substantially and can reach up to 50 times higher than national averages [13,14]. In agreement to this, in the current study, the point prevalence of smear positive TB in North Gondar Zone Prison was 1482.3 per 100,000 populations which was 9.1 times higher than the TB in the general population [15]. The prevalence of all forms of TB in the Amhara Regional State during the study period was 643 per 100,000 populations while the prevalence of smear positive TB was 168 per 100,000 populations. Therefore, the prevalence of TB among the prisoners was 8.8x times higher than in the region [16]. However, the prevalence in Gondar was lower than the report from Eastern Ethiopian prisons which was 1913/100,000 [11]. The difference could be due to the study design used as the Eastern Ethiopian study included those TB patients who are on TB treatment already and culture was employed to detect *M. tuberculosis* apart from microscopy. Studies from Zambia, Botswana, Russia and Georgia showed much higher prevalences [5,6,17,18]. On the other hand, lower prevalences were reported from prisons of some Asian and European countries, 568/100,000 in Thailand, 259/100,000 in Taiwan, 341/100,000 in Turkey and 215/100,000 in France [19-22]. The relatively lower prevalence in these countries could be due to a good TB control strategy and low TB incidence in the general population as well as in the prisons. High prevalence of TB in prisons could pose problems to the TB control in the general population as TB from inmates may spread through visitors, prison staff and discharged inmates into the community. Improving the TB control system in prisons could impact on the TB control in the community. Otherwise it could be a potential time bomb [23] for disrupting the recent progress made in TB control in the country.

The current study revealed that majority 20(76.9%) of the TB positive inmates developed the cough after they joined the prisons. The duration of cough most TB positive prisoners having was greater than 2 months. Even though there wasn’t any significant association between the duration of cough and TB positivity in this study, it could still show an extended lag time before patients get diagnosed and treated rendering the smear positive prisoners to transmit the infection to many others. This could be intensified by the nature of the cells shared by the inmates. The cells in the study area were poorly ventilated and gloomy and house hundreds of detainees (the mean number of inmates per cell was 333) who mix all day long with detainees from other cells in enclosed spaces. The lengthy stay of the inmates in the prison could have been rendering the prison to serve as a reservoir of TB transmission. The time left to stay in the prison for most of the TB positive inmates was more than a year which could further enhance transmission of TB. To make the matter worse, the prison’s health system in the study area was inadequate and poorly linked to the nearby public health institutions. This alarms the need for active periodic surveillance of TB and quarantine of the inmates with TB and linking them to the nearby public health institutions. Health education for inmates so as to reduce delay in seeking TB diagnosis and treatment could also help.

Co-infection with HIV in prisoners with active or latent TB is a well documented phenomenon [14,24,25] and this presents considerable diagnostic and management challenges to prison health systems. In the current study, a total of 19(7.6%) were found to be reactive for the HIV antibody test amongst of which 9 (47.4%) had TB co-infection. The prevalence of HIV infection in the TB infected inmates was calculated to be 34.6%. The HIV prevalence among the TB cases in the current study was higher than reports from Spanish and Tanzanian prisoners that showed 17.9% and 26% TB/HIV co-infection, respectively [7,26] while it was lower than for the Malawian prisoners who had HIV in up to 73% of the cases [8]. The immunosuppression could be one of the several factors that resulted in the high prevalence of TB in the study area. HIV infection and the associated immune suppression is a major risk factor for the development of active TB in those who acquire new *M. tuberculosis* infection or have latent *M. tuberculosis* infection. A report from SSA showed that HIV prevalence in prisons is more than twice the HIV prevalence of the general community [27]. Since TB and HIV epidemics are closely related, health services for prisoners must be aligned synergistically so that management with anti-TB drugs and antiretroviral therapy can be monitored effectively.

Apart from immunosuppression due to HIV, the high prevalence of TB in prisons is often related to prisoner-associated risk factors such as malnutrition [13,14,28]. In the current study, based on the BMI of the prison inmates, 46.2% of the TB positive inmates were undernourished (BMI < 18.5kg/m^2^). The under nutrition was found to be statistically associated with the TB positivity (*P = 0.025*). The under nutrition among the prisoners in the current study is much higher when compared to the prevalence of under nutrition in the general population in northwest Ethiopia, 12.9% [29]. Lack of proper nutrition, infection with TB, HIV and intestinal parasitoses, and presence of other co-morbidities might be the causes of the malnutrition. We suggest further studies to define the cause of malnutrition in the prison.

Several studies showed that among patients being retreated for TB because of initial treatment failure, default from initial treatment, or relapse following initial treatment, drug resistance was common and retreatment outcomes were generally poor [30,31]. Other reports showed that multi drug resistant (MDR) tuberculosis occurs 5–10 times more frequent in previously treated patients than among new patients [32]. In the present study, 10.8% of the inmates presented with history of previous treatment for TB. In addition to this, 19.2% of the TB positive inmates were previously treated for TB. Even though previous treatment wasn’t statistically associated with TB positivity, there is an increased risk for the inmates to develop drug resistant strain with a potential to spread to the general population. According to the nationwide anti-TB drug resistance survey conducted in 2005 in Ethiopia, the estimated MDR cases were 1.6% and 11.8% among newly and previously treated patients respectively [33]. A recent study in northwestern Ethiopia also indicated a 3.7% and 10.9% MDR TB prevalence in newly diagnosed and previously treated patients, respectively [34]. This could be alarming as the prison might have been a reservoir of MDR TB for various reasons. Factors that encourage the spread of TB in prisons also promote the spread of MDR forms. Therefore, further studies should be conducted to determine the prevalence of MDR-TB and act accordingly.

## Conclusion

There is high prevalence of TB in North Gondar Zone Prison with possible active transmission of TB among inmates. The prevalence of TB/HIV co-infection was also high. So, strong cooperation between prison authorities and the national tuberculosis control programmes is urgently required to develop locally appropriate interventions to reduce transmission, emergence of MDR TB and to help the control of the disease in the general population.

## Abbreviations

BMI: Body mass index; FM: Fluorescence microscopy; FNA: Fine needle aspiration; FNAC: Fine needle aspiration cytology; HIV: Human immunodeficiency virus; LED: Light emitting diode; MDR: Multi drug resistance; OR: Odds ratio; SSA: Sub Saharan Africa; TB: Tuberculosis; WHO: World health organization.

## Competing interests

The authors declared that there are no conflicts of interests.

## Authors’ contribution

BM was involved in the study conception and design, data collection, patient clinical evaluation, data analysis, and drafting the manuscript. BA, FA, MT, WT, and YB were involved in study design, data collection and reviewing the manuscript. While AK, and AM were involved in the study design and reviewing the manuscript. All the authors have read the manuscript, edited and approved.

## Pre-publication history

The pre-publication history for this paper can be accessed here:

http://www.biomedcentral.com/1471-2334/12/352/prepub
